# If it’s not one thing, HIF’s another: immunoregulation by hypoxia inducible factors in disease

**DOI:** 10.1111/febs.15476

**Published:** 2020-07-20

**Authors:** Ffion R. Hammond, Amy Lewis, Philip M. Elks

**Affiliations:** ^1^ The Bateson Centre Department of Infection Immunity and Cardiovascular Disease University of Sheffield UK

**Keywords:** HIF, hypoxia, infection, inflammation, innate immunity

## Abstract

Hypoxia‐inducible factors (HIFs) have emerged in recent years as critical regulators of immunity. Localised, low oxygen tension is a hallmark of inflamed and infected tissues. Subsequent myeloid cell HIF stabilisation plays key roles in the innate immune response, alongside emerging oxygen‐independent roles. Manipulation of regulatory proteins of the HIF transcription factor family can profoundly influence inflammatory profiles, innate immune cell function and pathogen clearance and, as such, has been proposed as a therapeutic strategy against inflammatory diseases. The direction and mode of HIF manipulation as a therapy are dictated by the inflammatory properties of the disease in question, with innate immune cell HIF reduction being, in general, advantageous during chronic inflammatory conditions, while upregulation of HIF is beneficial during infections. The therapeutic potential of targeting myeloid HIFs, both genetically and pharmacologically, has been recently illuminated *in vitro* and *in vivo*, with an emerging range of inhibitory and activating strategies becoming available. This review focuses on cutting edge findings that uncover the roles of myeloid cell HIF signalling on immunoregulation in the contexts of inflammation and infection and explores future directions of potential therapeutic strategies.

## Graphical abstract

Hypoxia‐inducible factors (HIFs) govern the cellular response to hypoxia, a hallmark of inflamed and infected tissues. In a generalised myeloid innate immune cell, HIF signalling influences cytokine production (and other signalling), lifespan, pathogen killing/control mechanisms and other cell behaviours including chemotaxis, polarisation (in macrophage) and degranulation (in neutrophils). HIFs are therefore attractive therapeutic targets for immunoregulation to reduce chronic inflammatory diseases and improve host defences against infections.

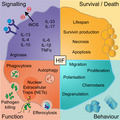

**Abbreviations**
AM: alveolar macrophages
ARG1: arginase 1
CCRCC: clear cell renal cell carcinoma
CoCl_2_
: cobalt chloride
COPD: chronic obstructive pulmonary disease
COVID‐19: coronavirus disease 2019
DMOG: dimethyloxalylglycine
HIF: hypoxia‐inducible factor
IL: interleukin
iNOS: inducible nitric oxide synthase
KO: knockout
Mm: Mycobacterium marinum
MPO: myeloperoxidase
Mtb: *Mycobacterium tuberculosis*
mTOR: mammalian target of rapamycin
NET: neutrophil extracellular trap
PAMP: pathogen‐associated molecular pattern
PHD: prolyl hydroxylase domain enzymes
PMA: phorbol myristate acetate
PPARγ: peroxisome proliferator‐activated receptor gamma
RA: rheumatoid arthritis
TB: tuberculosis
TLR: toll‐like receptor
VHL: Von Hippel–Lindau

## Introduction

**Fig. 1:**
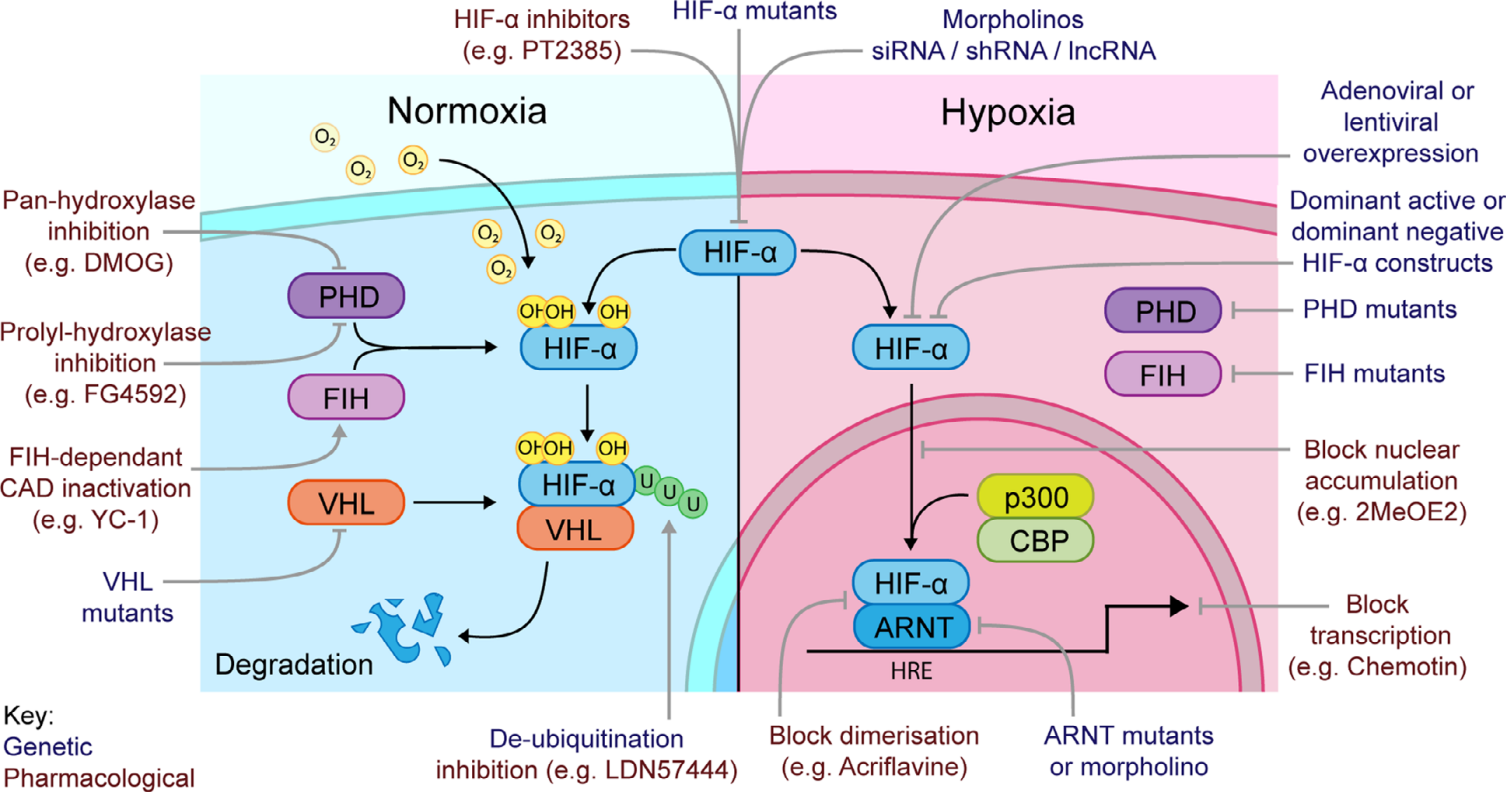
Genetic and pharmacological manipulation of HIF signalling. A schematic of intracellular regulation of the HIF‐α subunit, with normoxia on the left and hypoxia on the right. The therapeutic potential of targeting HIFs both genetically (orange text) and pharmacologically (green text) has been facilitated by an emerging range of both inhibitory and activating compounds/techniques (listed around the edge of the diagram). In normoxia (blue box), the HIF‐α subunit is targeted for degradation. This can be prevented genetically and pharmacologically via blocking initial hydroxylation by prolyl‐hydroxylases (PHDs) or factor‐inhibiting HIF (FIH), inhibiting Von Hippel–Lindau (VHL) binding to HIF‐α or reversing ubiquitination. In hypoxia (purple box), HIF‐α is stabilised and translocates to the nucleus to bind aryl hydrocarbon receptor nuclear translocator (ARNT) and cofactors p300 and CREB‐binding protein (CBP) to transcribe downstream targets. HIF‐α transcription can be inhibited via targeting HIF‐α directly using mutant animals/cells, by RNA‐based approaches (e.g. siRNA), or via blocking the nuclear accumulation of HIF‐α or the dimerisation of HIF‐α/ARNT complex.

Hypoxia‐inducible factors (HIFs) are master transcriptional regulators of the cellular response to hypoxia, that have influential roles in innate immune cell behaviour during inflammation and infections [1]. HIF activity is tightly regulated, both at the transcriptional and at the post‐translational levels [2, 3]. The major point of control is regulation of protein degradation of the HIF‐α subunits. In normal oxygen conditions (normoxia), the alpha subunits are hydroxylated by prolyl hydroxylase domain (PHD) enzymes leading to subsequent degradation by the proteasome (Fig. 1). PHD enzymes require oxygen for their enzymatic activity, and so when oxygen levels drop, PHDs become inactive and HIF‐α is stabilised, translocating to the nucleus to form its transcription factor complex that activates downstream genes. Multiple isoforms of the alpha subunit (HIF‐1α, HIF‐2α and HIF‐3α) contribute to an extra level of regulatory complexity [4, 5], and while HIF‐1α is the most widely characterised, important roles of HIF‐2α in immunity are emerging [6]. The HIF‐3α gene is the least understood of the isoforms, with alternative transcriptional start and splice sites contributing to different HIF‐3α variants that can have opposing functions [7]. Partially due to this complexity, the roles of HIF‐3α in immunity remain elusive.

HIF researchers were awarded the Nobel Prize in Physiology or Medicine in 2019 for their discoveries on the regulation of the cellular hypoxia response, with their medical research predominantly focusing on HIFs’ potential role as a therapeutic target to combat anaemia, due to HIF’s activating effect on red blood cell production [8]. Other blood cells, especially those of the myeloid lineage of innate immunity, are exquisitely sensitive to HIF modulation, due to their adaption to functioning in locations of low oxygen tension (e.g. wounds or infected tissues) [9]. The effects of HIF modulation on immune cells are wide‐ranging and context‐dependent. Over the past decade, HIFs have emerged as attractive targets for immunoregulation and immunotherapy due to their ability to profoundly influence immune cell behaviour and function, combined with roles in regulating inflammatory phenotypes in other cell types of the diseased tissue milieu. Here, we explore recent developments that illuminate HIFs’ roles in innate immunity and highlight HIF signalling components as therapeutic targets in multiple disease settings.

## HIFs’ effects on cellular innate immunity

HIFs are key regulators of myeloid innate immune cell function, influencing survival, migration and polarisation [9]. During infection and inflammation, HIF‐α is stabilised in immune cell populations, partially driven by the hypoxic tissue context of disease, alongside oxygen‐independent activation [10].

Neutrophils are the first innate immune cell responders to wounds or infections in many disease contexts and are exquisitely sensitive to low oxygen levels. HIF‐1α stabilisation increases the lifespan of neutrophils and their bactericidal capabilities in multiple experimental models [11, 12]. Neutrophil degranulation is an important process in pathogen control, but can cause extensive tissue damage when uncontrolled in chronic inflammatory diseases where tissue hypoxia is often a hallmark [13]. Human neutrophils in hypoxia have augmented degranulation, while neutrophils with stabilised HIF‐α, after treatment with the pan hydroxylase inhibitor, dimethyloxalylglycine (DMOG), interestingly do not have the same increased degranulation [14]. A dichotomy between physiological hypoxia and HIF stabilisation is also observed in neutrophil extracellular trap (NET) formation, a mechanism by which toxic components of the neutrophil (including histones, neutrophil elastase and granules) are ejected into the tissue microenvironment [15]. NETs have been implicated in the progression of multiple inflammatory conditions including chronic obstructive pulmonary disease (COPD), asthma and post‐COVID‐19 lung inflammation [16, 17, 18]. Neutrophils produce fewer NETs in hypoxic conditions, due to NET formation requiring the oxygen‐dependent respiratory burst [15]. Hypoxia is even able to ablate NET production when human blood‐derived neutrophils are exposed to the most potent NET inducing chemical, phorbol myristate acetate (PMA) [15]. However, HIF stabilisation in normoxia has been shown to increase neutrophil NET formation through mammalian target of rapamycin (mTOR) [19]. These observations could be especially important in understanding later stage COVID‐19 infection, where NET formation is predicted to play a major role in lung physiology [18].

Macrophages are a key cellular component of innate immunity, and like neutrophils, their function, migration and behaviours are influenced by hypoxia and HIF signalling [20]. Alveolar macrophages from myeloid‐specific VHL knockout (KO) mice possessing elevated HIF‐1α have increased pro‐inflammatory profiles, glycolytic enzyme activity and an abnormal, foamy morphology [21]. HIF’s roles in metabolic reprogramming of macrophages (and neutrophils), termed immunometabolism, are an emerging field of intense study in mice and humans 21, 22 that has been extensively reviewed elsewhere in recent years [23, 24, 25]. Not only do HIFs regulate macrophage pro‐inflammatory profiles, but they also play emerging roles in regulation of macrophage efferocytosis (clearance of apoptotic neutrophils) and a switch in macrophage polarisation towards ‘M2’‐like, anti‐inflammatory states that allows restoration of tissue homoeostasis. Macrophages from mice lacking HIF‐2α had increased efferocytosis compared to controls [26]. Myeloid HIF‐1α KO mice had attenuated anti‐inflammatory profiles, including a depressed arginase (ARG1) response, a key anti‐inflammatory macrophage enzyme [27]. HIF‐2α KO mice also have perturbed anti‐inflammatory macrophage cytokine profiles, with significantly lower IL‐6 (a liver protective cytokine) at mRNA and serum levels in models of liver injury [23], although in other disease situations IL‐6 is associated with pro‐inflammatory profiles [28]. Macrophage behaviours during wound repair can be improved using HIF‐directed therapies. In a mouse model of chronic kidney disease, the PHD inhibitor MK‐8617 increased the infiltration of macrophages into damaged muscle, improving muscle repair and reducing muscle atrophy [29]. Additionally, treatment of HIF‐α inhibitor YC‐1, in a mouse injury model, decreased numbers of pro‐inflammatory macrophages in scar tissue and reduced levels of pro‐inflammatory cytokines *in vivo* [30]. These studies indicate that macrophage HIF‐targeted therapies could allow a return to tissue homoeostasis after injury/inflammation.

## Dampening HIF in chronic inflammation

Chronic inflammation underpins diseases that are characterised by a hyperinflammatory profile with extensive immune cell‐induced tissue damage. High levels of hypoxia and HIF‐1α are associated with many of these conditions, including asthma, COPD, rheumatoid arthritis (RA), colitis and atherosclerosis [31, 32, 33, 34]. Furthermore, HIF signalling interplays with key inflammatory signalling pathways, including glucocorticoids, mTOR and arachidonic acid [35, 36, 37].

Targeting excessive HIF can be beneficial in the outcome of chronic inflammatory diseases. In a mouse model of asthma, suppression of HIF‐1α with YC‐1 reduced expression of IL‐5, IL‐13, myeloperoxidase (MPO) and inducible nitric oxide synthase (iNOS), which alleviated asthma symptoms [31]. In the highly inflammatory, granulomatous condition of sarcoidosis, downregulation of HIF‐1α through genetic or pharmacological means reduced pro‐inflammatory IL‐1β, IL‐17 and IL‐6 and improved disease outcomes [38]. However, downregulation of HIF‐1α can also be detrimental in some inflammation‐related conditions. HIF‐1α deficiency in B cells exacerbates collagen‐induced arthritis via impairment of IL‐10 and B‐cell expansion, highlighting a need to target‐specific immune cell subsets [39]. Stabilising HIF‐α subunits using hydroxylase inhibitors has been shown to be beneficial in some inflammatory conditions. DMOG suppresses the progression of periapical bone loss and attenuated inflammatory cell infiltration in apical periodontitis [40]. DMOG was also beneficial in a rat ischaemia model, reducing infarction size and increasing IL‐4‐ and IL‐10‐induced protection [41]. In a mouse model of multiple sclerosis, the dual peroxisome proliferator‐activated receptor gamma (PPARγ) and CB_2_ agonist, VCE‐004.8, was able to alleviate neuroinflammation and demyelination through inhibition of pro‐inflammatory cytokines, via activation of HIF [42]. These anti‐inflammatory effects of HIF are emerging and are likely to be a diseased tissue‐specific effect. DMOG treatment of macrophages directly increases expression of pro‐inflammatory factors such as iNOS and decreases anti‐inflammatory factors such as arginase [43]. This can be highly detrimental in inflammatory conditions. For example in an RA mouse model, HIF‐1α stabilisation with cobalt chloride (CoCl_2_), in combination with IL‐17, is associated with increased disease severity [44]. Stabilising HIF‐α in inflammatory diseases is therefore likely to be detrimental due to pro‐inflammatory effects, although tissue‐ and disease‐specific benefits may also occur.

Together these recent findings demonstrate the potential of HIF regulation to treat chronic inflammatory conditions through modulation of the myeloid component. However, treatments that target HIF can have pleiotropic effects, that differ in multiple cell types that are present in disease tissue contexts, and may require specific targeting.

## Upregulating HIF in infections

### Bacterial infections

**Fig. 2:**
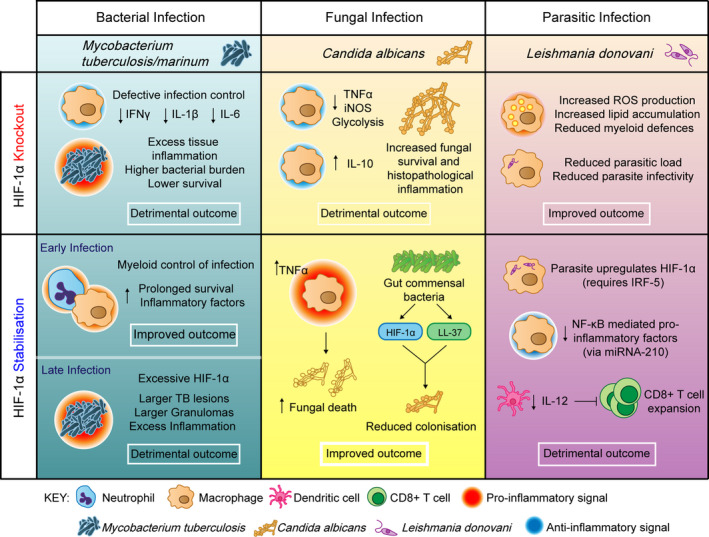
The contribution of HIF‐1α to infection outcomes. Figure showing the effects of HIF manipulation on bacterial, fungal and parasitic infection outcomes. Generally, HIF‐1α stabilisation is beneficial in the context of bacterial infections, for example *Mycobacterium tuberculosis/marinum* (blue column); however, the level of HIF‐1α must be carefully balanced, with too little or too much resulting in excess inflammation and detrimental infection outcome. HIF‐1α knockouts have decreases in IFNγ [77], IL‐1β [51] and IL‐6 [51] leading to excess tissue damage and lower survival [46]. Early HIF‐1α stabilisation increases inflammatory factors leading to myeloid control of infection [48, 49, 50], while excessive HIF‐1α can lead to prolonged inflammation and larger TB lesions in later stage disease [51]. In the context of the fungal infection *Candida albicans* (yellow column), HIF‐1α knockout decreases pro‐inflammatory factors while increasing anti‐inflammatory IL‐10 leading to increased fungal survival [55]. HIF‐1α stabilisation improves infection outcome, re‐arming the host inflammatory response leading to increased fungal death [55, 58]. In the parasitic infection *Leishmania donovani* (purple column), reducing HIF‐1α improves infection outcome, as the parasite itself upregulates host HIF‐1α, and stabilised HIF‐1α prevents CD8 + T‐cell expansion, exacerbating the infection further [62, 63, 64, 78]. Each infection context is distinct, requiring a tailored and controlled HIF‐α response that is not uniform across infections.

Due to the hypoxic microenvironment of many bacterial infections, combined with pathogen‐associated molecular pattern (PAMP)/TLR‐induced HIF‐α stabilisation, HIF has become increasingly associated with innate immune control of infections (Fig. 2).

Tuberculosis (TB) has the highest mortality of any single bacterial infection worldwide and has a complex relationship with HIF, in part due to the hypoxic centres of hallmark granuloma structures. *Mycobacterium tuberculosis* (Mtb) infection is associated with increased HIF levels in immune cells, with peripheral blood mononuclear cells (PMBCs) from human TB patients producing high levels of HIF‐1α, alongside pro‐inflammatory IL‐17 and IL‐1β when stimulated with Mtb strain HN878 compared with healthy donors [45]. Myeloid‐specific HIF‐1α KO mice have decreased survival upon Mtb infection and larger areas of lung inflammation, indicating that HIF‐1α plays a pivotal role in TB host defence [46]. Furthermore, HIF‐1α KO BMDMs have compromised lipid droplet formation, sites of eicosanoid synthesis that are required for host TB defence [47]. During early infection, HIF‐1α stabilisation is beneficial for innate immune control of TB. In a zebrafish TB model, using the fish‐adapted pathogen and close genetic relative of Mtb, *Mycobacterium marinum* (Mm), Hif‐1α stabilisation increased myeloid production of Il‐1β, vital for decreasing bacterial burden via neutrophil nitric oxide (NO) production [48, 49], alongside increasing pro‐inflammatory macrophage Tnfa production [50]. The HIF‐induced, host NO response has also shown to be important in a mouse model of TB infection, with HIF‐1α and iNOS in a positive feedback loop, balancing the inflammatory phenotype [51]. However, too much HIF‐1α during later stage TB infection can be detrimental, as HIF‐1α regulators prevent excess inflammation and host damage during active disease. IL‐17 has been shown to limit HIF‐1α expression in a murine model, with anti‐IL‐17 leading to an increased number of HIF‐1α‐positive macrophages and larger TB lesions [45]. Inhibition of HIF‐1α in these anti‐IL‐17 mice improved infection outcome, reducing granuloma size and reversing excess inflammation [45]. Together these recent data show critical roles of HIF‐1α in both innate immune cell TB control and in later stage granuloma pathogenesis.

Recent studies have added to the large bulk of evidence showing that HIF stabilisation is critical for the control of multiple bacterial infections [52, 53]. *Helicobacter pylori* infection of human gastric tissue stabilised HIF‐1α but not HIF‐2α, with HIF‐2α expression decreasing as disease severity increased [54]. HIF‐1α was shown to be crucial for bacterial killing of *Helicobacter pylori* as HIF‐1α KO neutrophils possessed 66% more surviving bacteria, and KO macrophages had 5 times more surviving bacteria compared to wild‐type [54]. HIF‐1α KO mice are also more susceptible to *Listeria monocytogenes* infection, possessing higher bacterial burdens and more severe pathological inflammation of the liver [55]. In a mouse model of uropathogenic *Escherichia coli* (UPEC), stabilising HIF‐1α using the hydroxylase inhibitor AKB‐4924 reduced bacterial burden by ~ 40% and attenuated the virulence of a hyperinfective UPEC strain [56]. Interestingly, activation of HIF‐1α in this way reduced the inflammatory profile in the murine bladder context, another example of HIF‐α stabilisation leading to reduced inflammation. The bactericidal properties of HIF‐1α stabilisation have now been moved into clinical settings, with promising results from CoCl_2_ containing bandages, that not only prevented bacterial survival and growth, but also promoted wound healing both *in vitro* and *in vivo* in a mouse infection model [57]. These recent studies highlight how host HIF‐1α could be stabilised to improve bacterial infection outcomes, particularly relevant due to rising occurrence of multidrug‐resistant bacterial strains.

### Fungal infections

Fungal infections are an emerging global concern, and recent studies have demonstrated important roles for HIF in their pathogenesis. Myeloid HIF‐1α KO mice were more susceptible to *Candida albicans* infection and have reduced NO and glycolytic activity [55]. Promoting HIF‐1α stabilisation with the hydroxylase inhibitor CoCl_2_ promoted fungal death *in vitro* (human macrophages) and *in vivo* (mouse) indicating a therapeutic potential for HIF‐1α manipulation in candida infection [55]. Activation of HIF‐1α has even emerged as being a natural mechanism by which commensal bacteria inhibit *Candida albicans* colonisation in the gut [58]. Infection of myeloid‐specific HIF‐1α KO mice with *Histoplasma capsulatum* had an increased anti‐inflammatory macrophage signature, increased fungal burden and decreased mouse survival, indicating important roles of HIF in infection control 59. However, alveolar macrophage (AM)‐specific HIF‐1α KO mice have comparable survival compared to WT, suggesting a contribution from other cells of the myeloid lineage [59]. These studies indicate an opportunity to stabilise HIF‐1α in hard‐to‐treat fungal infections. However, as some fungi are capable of producing and releasing their own proline hydroxylases, the effectiveness of PHD inhibitors as a therapy remains unclear [60].

### Parasitic infections

Some parasitic infections are able to circumvent host immunity by either failing to raise a pro‐inflammatory response or by mechanisms that actively reduce the host pro‐inflammatory response. *Trypanosoma brucei* suppresses host HIF‐1α through production of indolepyruvate, a ketoacid reported to be a cofactor of PHDs, promoting HIF‐1α degradation [61]. Therefore, in trypanosome infections it may be beneficial to stabilise HIF‐1α therapeutically. Conversely, *Leishmania donovani* infection increases levels of HIF‐1α, and silencing HIF‐1α in murine peritoneal macrophages reduced both parasitic load and infectivity at 24 hours postinfection [62]. BMDMs from HIF‐1α‐deficient mice were more resistant to *L. donovani* infection, expressed more ROS and were parasitised less compared to wild‐type [63]. However, a recent study has shown that *L. donovani*‐infected HIF‐1α‐deficient mice developed hypertriglyceridemia and lipid accumulation in splenic and hepatic myeloid cells, which impaired host defence [64]. Together these studies suggest that parasites are able to manipulate HIF‐1α levels in a variety of ways as part of their circumvention of the host immune response, creating an opportunity to target HIF‐1α to improve infection outcome that has yet to be fully explored.

## Future perspectives; targeting which HIF‐α, where?

Due to the complexity of innate immune contributions to inflammatory and infectious diseases, targeting the correct HIF proteins, in the correct cells, at the correct time may be important considerations for any therapeutic strategy.

Recent *in vitro* and *in vivo* model studies described here have largely focused on hydroxylase inhibition as the potential HIF modulation therapy. The most successful hydroxylase inhibitor in human trials is Roxadustat (otherwise known as FG‐4592). Roxadustat has undergone phase 3 clinical trials for chronic kidney disease‐induced anaemia and was able to successfully increase patient haemoglobin levels over a prolonged 8‐18 weeks period [65]. Other hydroxylase inhibitors used in clinical trials of various stages in recent years include the following: Molidustat [66], Vadadustat (or AKB‐6548) [67] and Daprodustat (GSK1278863) [68]. Recently, a study has shown that HIF‐α stabilisation can also be achieved by intrabody targeting of PHD2 [69]. HIF modulation via hydroxylase inhibition stabilises all HIF‐α isoforms, but there are emerging methods to modulate HIF‐α more directly. PT2385 is a HIF‐2α‐specific inhibitor that has been investigated in the first in‐human safety study and was shown to be well tolerated with a favourable safety profile for patients with clear cell renal cell carcinoma (CCRCC) [70] and may open the way for specific HIF‐α isoform stabilisation in future therapies against inflammatory diseases.

It is interesting to note that current clinical trials for anaemia and CCRCC have used oral doses of HIF‐targeting drugs, without delivery to specific cell types [65]. While there are no immediate indications that whole body HIF drug delivery is detrimental in the short term, this review highlights that in complex inflammatory conditions targeting the wrong HIF‐α isoform in the wrong cell type could be detrimental to disease outcome. For example, it was recently shown in a model of chronic inflammation of the cornea (Herpes Stromal Keratitis) that HIF‐1α was high in the myeloid lineage, whereas HIF‐2α was higher in epithelial cells, highlighting how different cell types in close proximity regulate HIF signalling differently and may need to be targeted separately in disease [71]. The potential of HIF‐1α stabilisation to contribute to inflammatory and infectious comorbidities has recently been explored in zebrafish models, where it was shown that although Hif‐1α stabilisation delays neutrophil inflammation resolution, a host‐protective effect against mycobacterial infection remains [72]. A potentially important step in immunoregulatory therapies would be cell‐specific targeting of HIF drugs in a timely way. Drug delivery methods, such as pH‐sensitive liposomes and liquid emulsion systems, have been used for timely delivery of HIF‐targeting drugs to specific tissues in animal models of disease [73, 74]. Emerging technologies, such as synthetic polymersomes that are preferentially taken up by innate immune cells, could be employed to deliver HIF‐targeted drugs to myeloid cells during infection and inflammation [75]. Gene therapy methods have also been explored, with nanoparticle delivery of siRNA for HIF‐1α used to genetically manipulate HIF in a murine model of hypoxic tumour growth [76].

In conclusion, hypoxia and HIF stabilisation are hallmark characteristics of progressed tissue inflammation and infection. The effects of HIF modulation on the innate immune response are profound, with recent findings illustrating that HIF’s effects can be complex and disease‐specific. Studies in *in vitro* and *in vivo* models indicate that HIF modulation during inflammation and infections may be promising therapeutic strategies against these hard‐to‐treat diseases. Subtle fine‐tuning of the innate immune system via HIF manipulation to achieve a balance between protection against pathogens and hyperinflammation will be an important consideration to develop future therapies that target host immune cells during diseases of inflammation and infection.

## Conflict of interest

The authors declare no conflict of interest.

## Author contributions

FRH, AL and PME cowrote the review.
